# Toward Precision Colorectal Cancer Screening: Integrating Clinical Risk Stratification, Molecular Biomarkers, and Artificial Intelligence

**DOI:** 10.3390/medicina62101875

**Published:** 2026-09-28

**Authors:** Sorinel Lunca, Stefan Morarasu, Gabriel Mihail Dimofte

**Affiliations:** 1 “Grigore T. Popa” University of Medicine and Pharmacy, 700115 Iasi, Romania; sorinel.lunca@umfiasi.ro (S.L.); mihail.dimofte@umfiasi.ro (G.M.D.); 2 2nd Department of Surgical Oncology, Regional Institute of Oncology, 700483 Iasi, Romania

**Keywords:** colorectal cancer, screening, precision medicine, risk stratification, molecular biomarkers, artificial intelligence

## Abstract

Colorectal cancer (CRC) screening reduces disease incidence and mortality through the detection and removal of precursor lesions and the earlier diagnosis of invasive cancer. However, most current programs remain predominantly age-based, applying broadly uniform tests and intervals to individuals whose underlying risk, lesion biology, screening adherence, and capacity to benefit may differ substantially. Advances in clinical prediction, molecular diagnostics, and artificial intelligence (AI) create opportunities to refine screening according to individual risk and clinical context. This narrative review examines the evolution of CRC screening from conventional population-based strategies toward a precision-screening framework comprising three complementary functions. Clinical risk stratification estimates background susceptibility using demographic, familial, behavioral, metabolic, medical, and previous-screening information. Molecular biomarkers provide biologically distinct information, ranging from inherited susceptibility to signals associated with prevalent colorectal neoplasia. AI may support multimodal risk integration, procedural lesion detection and characterization, and longitudinal reassessment as new clinical and screening information accumulates. These components should not be regarded as a mandatory sequential pathway or as interchangeable sources of evidence; rather, their value depends on the specific clinical question, predicted outcome, time horizon, and decision being supported. Current evidence shows that risk-adapted and multimodal approaches are feasible, but improved prediction or diagnostic performance does not necessarily translate into superior population outcomes. Most precision-screening strategies still lack prospective evidence demonstrating improvements in participation, diagnostic completion, advanced colorectal neoplasia yield, interval CRC, harms, resource use, equity, or cost-effectiveness compared with optimized established screening. The priority for translation is therefore not maximal technological integration, but selective use of additional information when it provides actionable incremental benefit while preserving the accessibility, effectiveness, and public-health strengths of organized screening.

## 1. Introduction

Colorectal cancer (CRC) remains a major cause of cancer-related morbidity and mortality despite being one of the malignancies most amenable to prevention and early detection. It is the third most frequently diagnosed cancer and the second leading cause of cancer-related death worldwide, accounting for more than 2.0 million new cases and approximately 918,000 deaths in 2024 [1]. The rising incidence of early-onset CRC among individuals younger than 50 years has also become an important concern, particularly in high-income countries [2,3].

CRC screening has substantially altered the natural history of the disease by enabling the detection and removal of premalignant lesions and the earlier diagnosis of invasive cancers [4,5,6,7]. Major guidelines recommend CRC screening, and US recommendations have lowered the starting age for average-risk screening from 50 to 45 years in response to the increasing disease burden among younger adults [8,9]. Despite differences in program organization, most strategies remain fundamentally population-based, with eligibility determined largely by age and screening intervals assigned according to broad risk categories. This approach provides substantial public-health benefit but assumes that individuals of similar age derive comparable benefit from the same screening modality and interval.

In reality, CRC risk reflects a complex interaction among age, sex, family history, lifestyle, metabolic health, and environmental exposures [2,10,11]. Screening-test performance is similarly heterogeneous. Multitarget stool DNA testing, for example, is more sensitive than the fecal immunochemical test (FIT) for CRC and advanced precursor lesions, but this gain is accompanied by lower specificity and a greater number of false-positive results [12,13]. Such trade-offs illustrate why no single screening modality is optimal for every individual.

Clinical risk-prediction tools, expanding molecular technologies [12,13,14,15], and artificial intelligence applied across the screening pathway [16,17] together create an opportunity to move beyond a purely age-based model. Their purpose is not to replace established screening modalities, but to refine who should be screened, when screening should begin, which test should be selected, and how frequently screening should be repeated. The field nevertheless remains fragmented: clinical risk models, molecular biomarkers, and artificial intelligence are generally evaluated independently, with limited evidence regarding how they should be integrated in practice.

This review traces the evolution of CRC screening from established population-based modalities toward an integrated precision-screening framework comprising three complementary layers: clinical risk stratification, molecular biomarkers, and artificial intelligence (Figure 1). It argues that the next phase of CRC prevention will depend on the responsible integration of these layers to deliver the most appropriate screening strategy to the right individual at the right time.

## 2. Materials and Methods

This narrative review was informed by targeted searches of PubMed, Embase, Scopus, and Web of Science, with the final search performed on 20 July 2026. No lower publication-date limit was applied, and only English-language publications were considered. Searches combined terms related to colorectal cancer screening with concepts including risk stratification, clinical prediction models, polygenic risk, stool- and blood-based biomarkers, DNA and RNA markers, microbiome-derived biomarkers, artificial intelligence, machine learning, computer-aided colonoscopy, individualized screening, and precision screening. Representative search combinations are provided in Supplementary Table S1.

Eligible sources included clinical guidelines, randomized and prospective screening studies, systematic reviews and meta-analyses, externally validated prediction studies, diagnostic-accuracy studies performed in screening-relevant populations, implementation and cost-effectiveness analyses, and original investigations directly relevant to the clinical application of precision CRC screening. Earlier landmark studies were retained when necessary to establish the biological or clinical foundations of current screening practice. Studies focused exclusively on symptomatic diagnosis, treatment, prognosis after established CRC, or technologies without a clear screening or surveillance application were not prioritized unless they informed an issue directly relevant to the proposed framework.

Evidence selection was guided by relevance to the review questions, study design, population representativeness, external validation, and clinical applicability rather than journal prestige. When findings conflicted, greater interpretive weight was given to randomized or prospective evidence, studies conducted in representative screening populations, externally validated models, and studies reporting clinically relevant outcomes rather than discrimination or diagnostic accuracy alone. Particular attention was given to participation, diagnostic completion, detection of advanced precursor lesions, downstream colonoscopy use, interval CRC, harms, resource use, and equity.

Reference lists of key guidelines, systematic reviews, and landmark studies were additionally examined to identify potentially relevant publications. References were checked against the original publication for study design, population, principal outcomes, and bibliographic accuracy before inclusion.

Because the objective was an integrative narrative synthesis rather than a systematic review, no formal PRISMA study-selection process, meta-analysis, or structured risk-of-bias assessment was performed. Accordingly, the review may be subject to selection bias inherent to narrative evidence synthesis, and the absence of formal study-level risk-of-bias scoring should be considered when interpreting comparisons across heterogeneous evidence sources.

## 3. Current Colorectal Cancer Screening: Achievements, Current Practice, and Remaining Challenges

CRC screening is one of the few cancer-prevention strategies capable of reducing both disease incidence—through the detection and removal of premalignant lesions—and mortality through earlier diagnosis. This dual benefit is made possible by the long preclinical interval during which conventional adenomas and serrated lesions can be identified and removed before progression to invasive cancer [10,18]. Randomized trials, long-term cohort studies, and organized screening programs have consistently demonstrated reductions in CRC incidence, mortality, or both [4,5,6,19,20,21,22]. Increasing recognition of substantial interindividual variation in CRC risk has nevertheless exposed the limitations of relatively uniform screening strategies and shifted attention toward more individualized, risk-adapted approaches. Throughout this review, “advanced precursor lesions” refers collectively to advanced conventional adenomas and clinically significant serrated precursor lesions, whereas “advanced colorectal neoplasia” refers to CRC together with advanced precursor lesions, unless a different definition is specified in an individual cited study. Advanced adenoma generally refers to a conventional adenoma ≥ 10 mm, with villous/tubulovillous histology, or high-grade dysplasia, unless otherwise defined in an individual study.

### 3.1. Evolution of Screening

The biological rationale for CRC screening can be traced to the adenoma–carcinoma sequence described by Fearon and Vogelstein, in which progressive genetic alterations drive the transition from normal epithelium through adenoma to invasive carcinoma [18]. Contemporary understanding has expanded to include the serrated neoplasia pathway, microsatellite instability, and broader molecular heterogeneity. Nevertheless, the central preventive principle remains unchanged: most CRCs develop through identifiable precursor lesions that can potentially be detected and removed before malignant progression [10].

The first robust evidence supporting population-based screening came from randomized trials of guaiac-based fecal occult blood testing, which demonstrated reductions in CRC mortality [4,19,20]. However, this approach was limited by modest sensitivity and its dependence on lesion bleeding. Endoscopic screening subsequently shifted the emphasis toward direct visualization and removal of colorectal lesions. The National Polyp Study demonstrated that colonoscopic polypectomy reduced subsequent CRC incidence [21], with later follow-up showing a reduction in CRC mortality [22]. Randomized flexible sigmoidoscopy trials likewise demonstrated durable reductions in CRC incidence and mortality [5,6], and 21-year follow-up of the UK trial confirmed that the protective effect of a single examination can persist for approximately two decades [23].

A clinically important perspective emerged from the NordICC trial, the first large randomized trial evaluating invitation to screening colonoscopy against usual care. In the initial report after a median follow-up of 10 years, only 42% of invited participants underwent colonoscopy. In the intention-to-screen analysis, invitation was associated with an 18% relative reduction in CRC incidence, whereas the reduction in CRC mortality was not statistically significant [24]. Adjusted per-protocol analyses estimated substantially larger reductions among individuals who underwent screening, although these estimates are more vulnerable to residual confounding because adherence is no longer randomized. The distinction generated considerable debate but is central to the interpretation of pragmatic screening trials: intention-to-screen analyses estimate the population effectiveness of offering a screening strategy, whereas per-protocol analyses seek to estimate the effect of actually undergoing screening. More recent 13-year follow-up has continued to demonstrate a reduction in CRC incidence and has reinforced the importance of both sufficient follow-up and screening uptake when interpreting the population impact of colonoscopy [25]. These findings support a fundamental principle of organized screening: the effectiveness of even a highly sensitive test depends not only on its intrinsic performance, but also on participation and completion.

The 2025 COLONPREV trial provides a complementary perspective on invitation-level effectiveness. Participation in any screening was higher among individuals invited to biennial FIT than among those invited to colonoscopy (39.9% vs. 31.8%). At 10 years, CRC mortality was 0.24% in the FIT group and 0.22% in the colonoscopy group, and invitation to FIT was non-inferior to invitation to colonoscopy within the prespecified non-inferiority margin. Together with NordICC, these findings emphasize that population effectiveness depends not only on intrinsic test performance, but also on participation, repeated adherence, and completion of diagnostic evaluation [26].

Organized-program infrastructure—including invitation systems, screening registries, quality assurance, and timely diagnostic follow-up—is therefore essential for translating test efficacy into population benefit [27]. The rising incidence of early-onset CRC has also prompted US recommendations to lower the starting age for average-risk screening from 50 to 45 years [8,9]. However, lowering the age threshold does not resolve the underlying limitation of chronological age as an imperfect surrogate for individual biological risk.

### 3.2. Established Modalities

No single screening test is optimal for every individual or healthcare system. Effective programs must balance sensitivity, specificity, safety, acceptability, accessibility, cost, available colonoscopy capacity, and the likelihood that participants will complete both the initial test and any required diagnostic follow-up. Table 1 summarizes the detection principles, principal strengths, limitations, and current clinical roles of the established screening modalities: guaiac-based fecal occult blood testing, fecal immunochemical testing, multitarget stool DNA and RNA testing, colonoscopy, flexible sigmoidoscopy, computed tomographic colonography, and colon capsule endoscopy.

Two considerations deserve emphasis beyond the comparisons presented in the table. First, greater single-round sensitivity does not necessarily translate into greater population benefit. Multitarget stool DNA testing detects more CRCs and advanced precursor lesions than FIT, but this gain is accompanied by lower specificity, more false-positive results, and greater downstream colonoscopy demand [12,13]. Second, the protective effect of colonoscopy depends substantially on examination quality. Adenoma detection rates vary meaningfully among endoscopists, and lower detection rates are associated with higher risks of interval CRC and CRC-related mortality [35,36].

### 3.3. Current International Recommendations

Major guidelines converge on a common principle: average-risk adults should be offered an evidence-based screening option, and every positive non-colonoscopic screening test should be followed by timely diagnostic colonoscopy. In the United States, the 2026 American Cancer Society (ACS) guideline continues to recommend initiation of average-risk CRC screening at age 45 and recognizes multiple preferred options, including annual FIT or high-sensitivity gFOBT, multitarget stool DNA or stool RNA testing every 3 years, colonoscopy every 10 years, computed tomographic colonography every 5 years, and flexible sigmoidoscopy every 5 years. Blood-based tests are recognized as an alternative only for individuals who decline or do not complete preferred stool-based or structural screening. Any positive non-colonoscopy screening test requires timely diagnostic colonoscopy to complete the screening process [34]. In Europe, the European Commission Initiative on Colorectal Cancer issued updated guidance in June 2025 recommending organized population-based screening for average-risk adults aged 50–69 years, with biennial FIT as the preferred approach and conditional continuation of screening up to age 74; endoscopy-based screening remains an alternative where FIT is not implemented [39].

Despite these organizational differences, screening eligibility and intensity remain determined largely by broad categories—including age, family history, hereditary syndromes, inflammatory bowel disease, and previous colorectal findings—rather than by the substantial variation in risk that exists within those categories. Across European countries, differences in the implementation, participation, and maturity of screening programs have occurred alongside marked variation in CRC incidence, mortality, and stage distribution during the screening era [40].

### 3.4. Toward Risk-Adapted Screening

Chronological age remains the organizing principle of current practice, but inherited susceptibility, lifestyle, metabolic health, previous screening findings, and procedural quality all provide information that age alone does not capture. At the same time, colonoscopy remains a finite resource and cannot support unlimited intensification across the entire population. Early randomized evidence also illustrates both the feasibility and the current limitations of risk-adapted screening. In the three-round TARGET-C trial, participants were randomized to one-time colonoscopy, repeated FIT, or a risk-adapted strategy in which individuals classified as high risk were referred for colonoscopy and those at lower risk underwent FIT. Participation was 42.4%, 99.3%, and 89.2%, respectively. Advanced colorectal neoplasia detection was 2.76% with colonoscopy, 2.17% with FIT, and 2.35% with the risk-adapted strategy; the latter did not demonstrate superiority over FIT (OR 1.09, 95% CI 0.88–1.35) [41]. The number of colonoscopies required to detect one advanced neoplasm was 15.4 with one-time colonoscopy, 7.8 with repeated FIT, and 10.2 with the risk-adapted strategy, illustrating the trade-off between screening yield and colonoscopy resource use [41]. These findings support the feasibility of population-level risk adaptation but also demonstrate that improved stratification does not necessarily translate into superior screening yield compared with an optimized FIT-based program.

## 4. Why Precision Colorectal Cancer Screening Is Needed

Organized CRC screening has substantially reduced disease incidence and mortality, establishing population-based screening as one of the most effective cancer-prevention strategies available [4,5,6,7,21,22,23,40,42]. Nevertheless, this success does not mean that the current paradigm is optimal. Screening recommendations are developed at the population level but applied to individuals whose underlying risk may differ substantially, even among those of the same age. This variation reflects differences in inherited susceptibility, lifestyle and environmental exposures, metabolic health, and the molecular pathways through which colorectal neoplasia develops [43,44,45,46,47,48,49,50,51]. The central limitation of current screening is therefore not the absence of effective tests, but the limited ability of largely uniform strategies to accommodate this biological and clinical heterogeneity.

Precision screening is best understood not as a new test, but as a shift in the underlying decision framework—from asking whether an individual has reached a predefined screening age to estimating that individual’s absolute probability of clinically significant colorectal neoplasia. Screening initiation, modality, and interval could then be adapted to the estimated level of risk, with the aims of improving diagnostic yield, allocating resources more efficiently, and identifying individuals who may benefit from earlier initiation, a more sensitive modality, or shorter screening intervals [11,52,53,54,55].

### 4.1. Biological Heterogeneity of Colorectal Cancer

CRC is increasingly understood as a heterogeneous group of diseases rather than a single biological entity. Although the conventional adenoma–carcinoma sequence predominates and provides the principal rationale for detecting and removing adenomatous precursor lesions [18,43], several distinct pathways contribute to colorectal carcinogenesis. These pathways are characterized by different molecular alterations, precursor lesions, anatomical distributions, and morphological features [43,47,56,57]. This heterogeneity has direct implications for screening because lesions arising through different pathways are not detected equally well by the same modalities.

A substantial proportion of CRCs arise through the serrated neoplasia pathway. Serrated precursor lesions are often flat or sessile, proximally located, and less likely to produce detectable bleeding, making them more difficult to identify through both fecal testing and colonoscopy [44,56,57]. Hereditary mismatch-repair deficiency, particularly Lynch syndrome, and chronic inflammatory carcinogenesis associated with inflammatory bowel disease represent additional biologically distinct pathways [46]. Dedicated CRC surveillance strategies are established for patients with colonic IBD [58]. Classification into Consensus Molecular Subtypes further demonstrates the molecular diversity of CRC, although these subtypes do not yet directly inform population-screening recommendations [47].

In practice, lesion morphology, anatomical location, bleeding behavior, and biological progression all influence detectability by FIT, colonoscopy, computed tomographic colonography, and molecular assays [44,59,60,61]. Consequently, apparently identical screening strategies may provide different levels of protection depending on lesion biology rather than chronological age alone.

### 4.2. Individual Variability in Colorectal Cancer Risk

Biological heterogeneity characterizes the disease, but equally important variation exists among the individuals being screened. Although guidelines classify most adults into broad risk categories, absolute CRC risk differs substantially among people of the same age because of variation in family history, lifestyle, metabolic health, inherited susceptibility, and previous colorectal findings [48,49,50,51,62,63,64]. Hereditary syndromes, including Lynch syndrome and familial adenomatous polyposis, confer markedly elevated risk and warrant syndrome-specific screening and surveillance pathways [45,65,66].

Modifiable factors—including obesity, physical inactivity, diets rich in red or processed meat, alcohol consumption, and smoking—are consistently associated with increased CRC risk and may act cumulatively [48,63,64,67]. Metabolic conditions, particularly type 2 diabetes and metabolic dysfunction-associated steatotic liver disease (MASLD), are also increasingly recognized as relevant contributors [50,51,68].

Chronic inflammatory conditions and previous colorectal findings—including advanced precursor lesions, such as advanced adenomas and clinically significant serrated lesions, as well as prior CRC—similarly predict future neoplasia and already influence surveillance recommendations [46,62,69]. Conversely, repeated negative screening results may indicate a lower near-term probability of advanced colorectal neoplasia than age alone would suggest, although the extent to which screening history should modify subsequent intervals remains incompletely defined. Taken together, these observations indicate that CRC risk exists along a continuum rather than within a small number of fixed categories, strengthening the rationale for individualized risk estimation.

### 4.3. Limitations of Age-Based Screening

Age-based eligibility is simple, epidemiologically grounded, and well suited to organized population-screening programs [8,9,24]. Most guidelines have therefore historically recommended that average-risk screening begin at a defined age, while advising earlier or more intensive screening for individuals with elevated baseline risk, and dedicated surveillance for those with established conditions or previous colorectal neoplasia [45,46,49,62,65,69,70]. Nevertheless, chronological age alone remains an incomplete surrogate for individual risk.

The most visible limitation is the increasing incidence of early-onset CRC, which has been documented internationally, although its magnitude and timing vary among regions [2,3,71,72,73,74]. Most patients with early-onset CRC do not have an identifiable hereditary cancer syndrome, demonstrating the limitations of relying solely on age, family history, or recognized inherited predisposition to identify younger individuals at risk [2,66,74]. In response to this trend, the American Cancer Society and the US Preventive Services Task Force lowered the recommended starting age for average-risk screening from 50 to 45 years [8,9]. This change broadens eligibility but remains an adjustment of the same population-based framework rather than a transition toward individualized risk assessment.

Age may also overestimate or underestimate risk within the screening-eligible population. A healthy 50-year-old with a favorable lifestyle may have a lower near-term probability of advanced colorectal neoplasia than another individual of the same age with obesity, type 2 diabetes, and a long smoking history but no formally recognized high-risk condition. Yet age-based frameworks may distinguish their screening needs only minimally. Chronological age also fails to capture how risk evolves as body weight, smoking exposure, metabolic health, family history, and previous screening findings change over time [11,48,50,51,53].

Variation in test performance further compounds this limitation. FIT is substantially more sensitive for CRC than for advanced precursor lesions, and serrated lesions may be particularly difficult to detect because they often bleed less consistently [28,59,60]. The protective effect of colonoscopy likewise depends on bowel preparation, completeness of examination, and adenoma detection rate, which varies meaningfully among endoscopists [35,36,61]. Interval CRCs may arise from missed lesions, incomplete resection, or rapidly progressing tumor biology [75,76]. Lowering the screening starting age may also substantially increase colonoscopy demand [77], while modeling studies suggest that risk-adapted strategies may improve cost-effectiveness, although prospective evidence of clinical benefit remains limited [52,54].

These limitations do not diminish the value of organized screening programs, which remain the standard of care. Rather, they support treating age as one component of a broader, multidimensional assessment of individual risk, screening history, and capacity to benefit.

### 4.4. The Rationale for Precision Screening

Growing recognition of the biological and clinical heterogeneity of CRC shifts the objective of screening from age-based eligibility alone toward estimation of each individual’s probability of clinically significant colorectal neoplasia. Precision screening is therefore a decision-making framework rather than a new test. It combines multiple sources of information to refine screening initiation, modality, and interval while preserving the established benefits of population-based strategies [11,52,53,54,55].

Colonoscopy, FIT, stool DNA testing, and blood-based assays are not replaced, but used within a more individualized framework based on the principle that individuals with substantially different levels of risk may not derive equal benefit from identical recommendations. Current guidelines already apply this logic to hereditary syndromes, inflammatory bowel disease, family history, and previous colorectal neoplasia [45,46,49,62,65,69,70]. Precision screening extends the same principle to the broader population.

**From Population Screening to Risk-Adapted Precision.** Three related levels of increasing individualization can be distinguished conceptually. The first is population-based screening, which relies predominantly on age and remains the cornerstone of organized programs [8,9,24,70]. The second is risk-adapted screening or surveillance, in which recognized high-risk conditions—including hereditary syndromes, inflammatory bowel disease, family history, and previous neoplasia—justify earlier initiation, more intensive testing, or shorter follow-up intervals [45,46,49,62,65,69]. The third is precision screening, which incorporates clinical characteristics, metabolic health, lifestyle, polygenic risk, molecular biomarkers, microbiome-derived signals, screening history, and computational prediction to refine recommendations according to estimated risk and expected benefit [11,28,53,78,79,80,81,82,83,84,85,86,87]. Each level retains the strengths of the preceding one while adding further discriminatory information. Precision screening should therefore be understood as an evolution of established practice rather than its replacement.

**The Five Fundamental Questions of Precision Screening.** In practice, precision screening seeks to refine five decisions: who should enter or remain in screening; when screening should begin; which modality should be selected; how frequently testing should be repeated; and when screening or surveillance intensity should change. Eligibility and timing may increasingly reflect estimated absolute risk rather than age alone. Test selection may involve FIT, colonoscopy, stool-based molecular testing, computed tomographic colonography, or blood-based assays matched to the individual’s risk profile, prior screening findings, patient preference, and healthcare-system capacity. Screening intervals may also eventually vary according to baseline risk and previous findings rather than remaining uniform across the population. Because risk evolves over time, new clinical conditions, changes in family history, biomarker results, and accumulated screening findings may justify reassessment, although many such adaptations still require prospective validation.

**Current Evidence.** Multivariable clinical models generally provide modest improvement in the prediction of advanced colorectal neoplasia compared with age alone, establishing proof of concept but not yet a universally applicable clinical solution [11,55,88,89]. Polygenic risk scores can identify subgroups with substantially different risks despite similar demographic characteristics [53,78,79,80], although ancestry-related transportability remains unresolved. Molecular technologies extend risk assessment beyond demographic and clinical variables, but many still require prospective evaluation in representative screening populations [81,82,83,90,91,92,93,94]. AI may contribute by integrating these heterogeneous data and supporting longitudinally updated risk estimates [84,85,86,87,95,96]. However, external validation and prospective evidence of clinical utility remain uneven. Precision screening should therefore currently be regarded as an emerging strategy rather than an established standard of care.

**Clinical Implications.** The transition toward precision screening will probably be gradual. Validated clinical risk models may first complement existing guidelines, while molecular biomarkers, polygenic risk scores, and AI-based tools are incorporated as supporting evidence matures. The objective is not indiscriminately more screening, but a more favorable balance of benefit, harm, burden, and resource use for each individual’s evolving risk profile.

## 5. Clinical Risk Stratification: The First Layer of Precision Colorectal Cancer Screening

The transition from population-based to precision CRC screening begins most practically with clinical risk stratification. Clinical risk stratification provides a pragmatic entry point for precision screening because demographic, familial, lifestyle, metabolic, and prior-screening information is routinely available and can be combined to estimate an individual’s probability of CRC or advanced colorectal neoplasia [11,48,55,63]. Within the framework proposed here, clinical risk stratification represents the conceptual layer concerned with baseline susceptibility and does not imply that risk assessment must precede every other screening component.

Clinical risk stratification extends beyond the identification of isolated risk factors. Its purpose is to combine multiple determinants into an individualized estimate that may inform screening initiation, modality and interval [11,55,86]. No single variable, including age, predicts colorectal neoplasia with sufficient accuracy for individualized decision-making. However, combining several determinants generally provides modest improvement in discrimination beyond age-based eligibility alone [11,55,89].

### 5.1. From Risk Factors to Risk Prediction

A risk factor describes an association observed at the population level, whereas a prediction model combines multiple variables to estimate an individual’s probability of a specified outcome within a defined time horizon. Its clinical value depends not only on discrimination, but also on calibration, external validity, feasibility, and the ability to inform a meaningful change in management [11,55].

Age illustrates this distinction clearly. A 50-year-old woman with a favorable lifestyle may have a substantially different probability of advanced colorectal neoplasia from a 50-year-old man with obesity, type 2 diabetes, and a long smoking history, even though both meet the same age-based eligibility criterion [55,89,97]. Risk factors with modest individual effects may therefore become clinically informative when considered in combination, which is the central premise of multivariable prediction.

The Imperiale scoring system illustrates this principle. Using age, sex, waist circumference, cigarette smoking, and family history of CRC, it stratified asymptomatic adults undergoing initial screening colonoscopy into four risk groups. In the derivation cohort, the prevalence of advanced colorectal neoplasia increased from 1.92% in the very-low-risk group to 24.9% in the high-risk group; corresponding values in the validation cohort were 1.65% and 22.3%, respectively [88]. Although such a score does not provide diagnostic certainty, it identifies clinically meaningful differences in baseline probability across individuals.

Clinical risk should also be viewed as dynamic rather than fixed. Body weight, smoking exposure, metabolic health, previous screening findings, and recorded family history may change over time, suggesting that risk estimates may require periodic reassessment rather than a single evaluation at screening entry [11,55]. Most current models achieve only modest-to-moderate discrimination and show variable performance across external populations [11,89,97]. Nevertheless, perfect prediction is not required for clinical usefulness, provided that a model is well calibrated, externally validated, feasible to implement, linked to predefined actions, and shown to provide net benefit compared with existing practice.

Clinical risk stratification should therefore be regarded neither as a replacement for screening nor as complete personalization, but as the first conceptual layer of precision screening.

### 5.2. Established Clinical Determinants

Clinically useful determinants should be reproducibly associated with CRC or advanced colorectal neoplasia, measured reliably, and feasible to collect in routine practice. Age remains the strongest conventional predictor, although its limitations are underscored by the rising incidence of early-onset CRC [55,64]. Men generally have a higher prevalence of colorectal adenomas and CRC than women of comparable age [55,88,98].

Family history is among the most clinically accessible determinants of CRC risk. Having an affected first-degree relative is associated with an approximately twofold increase in risk, although the magnitude varies according to the number of affected relatives, their age at diagnosis, and the presence of additional familial clustering [49]. Family history therefore provides information not captured by age alone and may justify earlier initiation, shorter intervals, or selection of a more intensive screening strategy. Its usefulness, however, depends on accurate reporting and periodic updating, because family-history information may be incomplete at the initial assessment or change as additional relatives are diagnosed.

Among potentially modifiable determinants, excess adiposity—particularly central or visceral adiposity—is consistently associated with CRC risk and is incorporated into some advanced colorectal neoplasia prediction scores [48,63,88]. Type 2 diabetes, smoking, alcohol consumption, and diets high in red or processed meat are also associated with increased risk, although several of these exposures are difficult to measure consistently in routine practice [48,50,63]. Long-term aspirin use has been associated with lower CRC incidence in selected populations, but its preventive application is constrained by bleeding risk and should not be considered a general screening determinant [48,63]. Metabolic dysfunction-associated steatotic liver disease is an emerging candidate predictor, although residual confounding by obesity and diabetes makes it premature to regard MASLD as an independent indication for altered screening [68].

These determinants frequently cluster and interact rather than operate in isolation. The practical objective is therefore not to compile an ever-expanding catalogue of risk factors, but to identify combinations that improve prediction and can be linked to actionable screening decisions through externally validated multivariable models rather than isolated thresholds [11,55,89].

### 5.3. Clinical Risk-Prediction Models

Clinical risk-prediction models differ in their intended population, predicted outcome, time horizon, and clinical purpose [11,55]. Some estimate the probability of prevalent advanced colorectal neoplasia at screening colonoscopy, whereas others estimate future incident CRC over several years or across the life course. A further group combines clinical, lifestyle, environmental, and genetic information to estimate the age at which an individual reaches a predefined population-risk threshold. These outcomes are not interchangeable, and model performance must therefore be interpreted in relation to the specific decision the model is intended to support.

Age, sex, family history, smoking, and measures of adiposity are among the most frequently included predictors [11,55,89]. Discrimination is generally modest to moderate, with reported AUCs commonly ranging from approximately 0.60 to 0.75 [55,89]. Such performance should be judged by whether a model meaningfully separates groups with different levels of risk rather than by the standards applied to a stand-alone diagnostic test.

The Imperiale model demonstrates clinically meaningful risk separation using readily available variables, although its performance and calibration require evaluation before application in other populations [88]. The Asia-Pacific Colorectal Screening score uses age, sex, first-degree family history of CRC, and smoking status and identified an approximately fourfold difference in advanced colorectal neoplasia prevalence between high- and average-risk groups [99]. It has subsequently been evaluated in external populations, with variable discrimination and calibration [100,101]. Additional predictors may improve performance in selected cohorts, but often at the expense of simplicity and transportability. Combining a clinical risk score with stool-based molecular testing may further improve the detection of advanced colorectal neoplasia [102].

A different family of models estimates future CRC risk over a longer time horizon. The QCancer 10-year risk algorithms illustrate the feasibility of estimating future cancer risk from routinely collected primary-care data. The bowel-cancer model was developed using QResearch electronic health records and estimates 10-year risk using variables including age, smoking, alcohol use, family history of bowel cancer, ulcerative colitis, previous colonic polyps, diabetes, and selected prior cancers [103]. Importantly, this future-risk model is distinct from earlier symptom-based QCancer algorithms designed to estimate the probability of an already existing but undiagnosed cancer. The absolute-risk model developed by Jeon and colleagues combined lifestyle, environmental, and genetic factors to estimate the age at which an individual reaches a conventional population-risk threshold for screening. The estimated starting age differed by a decade or more across combined risk profiles [53].

An AUC of 0.60–0.75 is insufficient for individual diagnosis but may still be useful for population stratification if the model separates groups with clinically meaningful differences in risk [55,89]. The Imperiale and APCS scores illustrate that several-fold differences in advanced colorectal neoplasia prevalence can emerge despite only modest discrimination [88,99]. Calibration should be evaluated alongside discrimination, while decision-curve analysis can assess whether use of the model is likely to provide clinical net benefit across relevant thresholds [104,105].

A model becomes clinically useful only when predefined risk categories are linked to explicit actions, such as earlier screening initiation, selection of colonoscopy rather than a non-invasive test, prioritization of colonoscopy when capacity is constrained, or modification of screening intervals. These actions should preserve informed patient preference and be evaluated prospectively against existing practice. Table 2 summarizes the representative models discussed above.

### 5.4. From Prediction Models to Clinical Practice: Current Challenges

A model that performs well in its development study is not necessarily ready for clinical implementation. External validation is required to determine whether discrimination and calibration are preserved in new individuals, whereas transportability concerns whether the model remains useful across different populations, settings, and healthcare systems. When several CRC risk models were evaluated head-to-head in the UK Biobank, their performance varied meaningfully within the same external cohort [97]. Transparent reporting according to TRIPOD and critical appraisal using PROBAST are therefore essential for identifying potential bias related to participant selection, predictor and outcome definitions, sample size, missing data, overfitting, and analytical methods [106,107].

Most existing models estimate risk from a single assessment, whereas an individual’s estimated risk may change as metabolic health, lifestyle, family history, and screening findings evolve. Periodic reassessment is useful, however, only when movement across a risk threshold leads to a predefined change in management. Many risk categories are derived statistically rather than linked to evidence that a different screening strategy improves outcomes. Thresholds should therefore be connected to explicit clinical actions and evaluated through prospective impact studies.

Whether the underlying model is simple or computationally complex, implementation should impose minimal workflow burden and generate outputs that are interpretable and actionable. Automation should support rather than replace clinical judgment and shared decision-making.

Participation, cost-effectiveness, and equity are closely interconnected. Economic evaluations indicate that the value of risk-stratified screening is highly sensitive to participation, implementation costs, screening thresholds, and the intensity of the resulting recommendations [52,54,108]. Conclusions regarding cost-effectiveness have differed across studies. Polygenic risk-stratified screening was not cost-effective under the base-case assumptions of some analyses [52], whereas other evaluations found strategies based on phenotypic factors, genetic risk, or family history potentially favorable under different assumptions [54,108]. Importantly, economic conclusions also depend on assumptions regarding adherence across repeated screening rounds, completion of diagnostic colonoscopy after a positive test, assay costs, surveillance intensity, and whether additional testing displaces or supplements an established screening strategy. Cost-effectiveness estimates should therefore not be transferred directly across healthcare systems without considering these context-specific assumptions.

Equity must also be considered, because models developed in insufficiently diverse populations may perform less well in underrepresented groups, while polygenic scores derived predominantly from European-ancestry cohorts may have reduced transferability [78,79,80].

Routine implementation therefore requires external validation and, where necessary, recalibration in the intended population; explicit alignment of the predicted outcome and time horizon with the clinical decision; actionable thresholds evaluated through decision analysis; prospective comparison with current screening strategies; and integration into clinical workflows with minimal additional burden.

Clinical risk stratification should be regarded as a foundational but incomplete component of precision screening. Its predictive capacity is constrained by the indirect relationship between clinical characteristics and the actual presence or biological activity of early neoplasia. Molecular biomarkers may complement this first layer by providing more direct evidence of disease-related biological change.

### 5.5. Clinical Implications

The most realistic near-term role of clinical risk stratification is to complement rather than replace established screening programs. Validated models may help identify individuals who could benefit from earlier screening, consideration of colonoscopy as the initial modality, or more intensive support to complete screening and diagnostic follow-up. Individuals with persistently low estimated risk may eventually be candidates for less intensive strategies, although such de-intensification requires prospective confirmation of safety.

## 6. Molecular Biomarkers: The Second Layer of Precision Colorectal Cancer Screening

Clinical risk stratification estimates susceptibility using demographic, familial, behavioral, metabolic, and medical characteristics, but these variables remain indirect: they estimate the probability of colorectal neoplasia without determining whether disease is already present. Individuals with similar clinical risk profiles may nevertheless harbor markedly different molecular alterations, precursor lesions, or occult malignancies [90,91,109]. Molecular biomarkers seek to address this gap by detecting biological signals—including occult bleeding, DNA methylation, somatic mutations, chromosomal instability, circulating tumor-derived nucleic acids, protein signatures, microbial dysbiosis, and metabolic alterations—that provide more direct evidence of disease-related biological change than clinical risk factors alone [91,92,93,94,110].

Molecular biomarkers should therefore be regarded as complementary to clinical risk stratification rather than as competing alternatives. Clinical variables estimate background susceptibility, whereas biomarkers seek evidence that neoplastic or cancer-associated biological processes may already be present [90,91,111]. A screening biomarker must be reproducible, feasible, and evaluated according to its intended clinical role—whether as a replacement, add-on, triage, or rescue test. For biomarkers intended to preserve the preventive function of screening, detection of clinically significant precursor lesions is as important as sensitivity for invasive CRC.

Clinical utility must also be demonstrated prospectively, because performance observed in retrospective case–control studies may decline when assays are evaluated in representative screening populations with lower disease prevalence and a broader spectrum of lesions [112]. Population benefit further depends on specificity, participation, testing interval, completion of diagnostic follow-up, cost, and downstream colonoscopy demand rather than on sensitivity alone [111,113].

The molecular landscape has expanded rapidly. Stool-based assays remain the most established biomarker tools, whereas blood-based tests, microbiome-derived signatures, metabolomic profiles, and multimodal panels are undergoing increasingly intensive clinical evaluation.

### 6.1. Why Molecular Biomarkers Are Needed

Molecular alterations associated with colorectal carcinogenesis—including APC inactivation, KRAS activation, TP53 loss, mismatch-repair defects, aberrant DNA methylation, and chromosomal instability—may accumulate long before clinical symptoms develop [18,114,115]. Molecular biomarkers are intended to exploit this preclinical window by detecting disease-associated biological signals while neoplasia remains asymptomatic and potentially preventable or treatable.

This opportunity is particularly important in average-risk screening populations, where disease prevalence is low and even small changes in sensitivity or specificity can have substantial consequences for false-positive findings, cost, and downstream colonoscopy demand.

Not all biomarkers serve the same purpose. FIT detects occult bleeding and functions as a biological screening marker, whereas other assays target tumor-associated DNA alterations, circulating cell-free DNA, microbial dysbiosis, extracellular vesicles, metabolomic profiles, or protein-based classifiers. These approaches interrogate different biological processes and may therefore have different clinical roles.

Because screening aims both to prevent CRC and to detect invasive disease at an early stage, performance should be reported separately for invasive CRC and clinically significant precursor lesions [111]. Population benefit also depends on repeated participation and timely completion of diagnostic colonoscopy after a positive non-invasive test [29,116].

### 6.2. Stool-Based Biomarkers: The Most Established Molecular Layer

Stool-based biomarkers have the strongest accumulated evidence for population CRC screening. Colorectal neoplasms may release blood, exfoliated epithelial cells, DNA, RNA, proteins, and microbial products into the intestinal lumen, permitting non-invasive sampling of biological material originating directly from the target organ [117]. The quantity and composition of this material vary according to lesion size, morphology, location, and biological characteristics, but stool testing can identify disease-associated signals even when a lesion remains confined to the colorectal mucosa.

Although FIT is an occult-blood test rather than a molecular assay, it is among the most established forms of biomarker-based screening. Repeated FIT screening is associated with reduced CRC mortality and limits diagnostic colonoscopy to individuals with a positive result [29,30,31]. Because FIT measures fecal hemoglobin quantitatively, screening programs can adjust the positivity threshold according to available colonoscopy capacity, although higher thresholds reduce referral demand at the cost of lower sensitivity.

Its principal limitation is that bleeding is an indirect and variable consequence of neoplasia. FIT has greater sensitivity for invasive CRC than for advanced precursor lesions; detection of serrated lesions is particularly limited because these lesions tend to bleed less consistently [28,44,60].

Multitarget stool DNA testing combines detection of exfoliated neoplasia-associated DNA alterations, including methylation markers and KRAS mutations, with measurement of fecal hemoglobin. This biological complementarity produces higher single-round sensitivity than FIT for CRC and advanced precursor lesions, but at the cost of lower specificity, more false-positive results, and greater downstream colonoscopy use [12]. An additional advantage is that lesions that do not bleed consistently may nevertheless release abnormal DNA into the stool, although this benefit must be weighed against higher cost, more complex sample processing, and increased demand for diagnostic colonoscopy. Next-generation platforms have further refined marker selection and test performance [13].

Multitarget stool RNA testing represents a newer stool-based molecular approach. The assay combines RNA-transcript signals with FIT and selected clinical information and demonstrated higher sensitivity than FIT for CRC and advanced adenomas in prospective validation [32]. It was subsequently incorporated into the 2026 American Cancer Society guideline as a preferred stool-based option at a 3-year interval [34]. However, its evidence base remains less mature than that of FIT or established stool DNA testing, particularly regarding repeated-round performance, long-term outcomes, and real-world implementation [33].

### 6.3. Blood-Based Biomarkers: Expanding Accessibility

Blood collection is already embedded in routine clinical care and may be more acceptable to individuals who decline stool collection or colonoscopy. The potential value of blood-based screening therefore lies partly in its capacity to improve participation, not solely in diagnostic accuracy.

Early-stage neoplasms generally release only small quantities of tumor-derived material into the circulation, contributing to the substantially greater sensitivity of current blood-based assays for invasive CRC than for advanced precursor lesions. Their performance should consequently be evaluated against two distinct objectives: early cancer detection and cancer prevention through identification of clinically significant precursor lesions.

Methylated SEPT9 was the first blood-based marker to undergo large prospective evaluation. The PRESEPT study established proof of principle that a plasma-based assay could detect a proportion of CRCs in asymptomatic screening participants, although sensitivity for precursor lesions was limited [118].

More recent multimodal assays combining methylation, mutation, and fragmentomic signals have achieved higher sensitivity for invasive CRC. In the prospective ECLIPSE study, a cell-free DNA blood test demonstrated 83% sensitivity for CRC and 90% specificity for advanced neoplasia, but only 13% sensitivity for advanced precursor lesions [14]. These findings illustrate the central trade-off of blood-based screening: such assays may improve early cancer detection and increase participation among individuals unwilling to complete established tests, but they could reduce the preventive effectiveness of screening if they replace more precursor-sensitive stool-based strategies among people who would otherwise adhere to them.

Importantly, regulatory authorization, guideline positioning, and commercial availability represent different levels of evidence and should not be conflated with demonstrated population benefit. The 2026 ACS guideline recognizes blood-based CRC screening as an alternative for individuals who decline or do not complete preferred screening, but does not classify it as a preferred strategy because of lower sensitivity for advanced precursor lesions and stage I CRC and uncertainty regarding repeated-round effectiveness [34].

Circulating RNA and microRNA signatures and extracellular-vesicle biomarkers remain at an earlier stage of development. Systematic reviews have reported promising diagnostic performance [119,120], but the underlying evidence is still derived largely from retrospective and case–control studies that may overestimate performance in representative screening populations. These approaches should therefore remain classified as investigational rather than clinically established.

### 6.4. Microbiome and Metabolomic Biomarkers

CRC has been associated with recurring alterations in gut microbial composition, including enrichment of organisms such as *Fusobacterium nucleatum* and pks-positive *Escherichia coli*. Some of these changes may contribute to carcinogenesis, whereas others may arise secondarily from the tumor microenvironment or other disease-related processes.

Multiorganism signatures generally show better diagnostic performance than individual microbial markers, but reproducibility across populations remains a major challenge because the microbiome is influenced by diet, geography, medication exposure, sampling methods, sequencing platforms, and bioinformatic pipelines [121].

Metabolomic biomarkers provide a complementary functional perspective by capturing disease-associated changes in host and microbial metabolism. However, they remain limited by heterogeneity in specimen type, analytical platform, normalization, and validation methods [122,123]. Microbiome- and metabolomic-derived signatures may therefore ultimately be more useful as components of composite biomarker panels than as stand-alone screening tests.

### 6.5. Multimodal Biomarker Panels

No single biomarker captures the full biological heterogeneity of CRC, making the combination of biologically complementary signals a logical strategy, as already illustrated by multitarget stool DNA testing. Integration should be driven by genuine incremental value rather than by the indiscriminate accumulation of variables. Each additional component should justify its cost and complexity through improved sensitivity, specificity, calibration, clinical net benefit, or feasibility in the intended screening setting.

The optimal combination will depend on the clinical role of the test, the population being screened, available resources, and downstream colonoscopy capacity. Artificial intelligence may play an increasing role in integrating and interpreting multimodal signals, particularly when relationships among variables are nonlinear or high-dimensional. Nevertheless, a simpler and more affordable panel with slightly lower diagnostic performance may produce greater population benefit if it is easier to implement, more acceptable to participants, and more cost-effective.

### 6.6. From Biomarker Performance to Clinical Utility

The development pathway for a screening biomarker should progress from analytical validation to clinical validation in representative screening populations, followed by demonstration of clinical utility and, ultimately, evidence that its use improves patient- or population-level outcomes. Many candidate biomarkers do not advance beyond early case–control studies, in which comparisons between patients with established cancer and healthy controls may produce overly optimistic performance estimates because of spectrum bias [112].

Sensitivity should be reported separately for invasive CRC and advanced precursor lesions, because a single combined estimate may obscure clinically important differences between cancer detection and cancer prevention, as illustrated by the marked difference between CRC and advanced-precursor sensitivity in the ECLIPSE study [14].

The intended role of a biomarker—replacement of an established test, addition to an existing pathway, triage before colonoscopy, or rescue testing for individuals who decline standard modalities—should be defined before its performance is interpreted. Modeling studies indicate that the population effect of a blood-based test depends substantially on whether it supplements established screening or substitutes for a more precursor-sensitive modality [111,113]. Cost-effectiveness analyses further suggest that blood-based tests require a favorable combination of test cost, specificity, participation, screening interval, and completion of diagnostic follow-up to approach the population benefit of established stool-based strategies [124].

### 6.7. Clinical Implications

Stool-based biomarkers currently provide the strongest evidence for prevention through the detection of both CRC and clinically significant precursor lesions. Blood-based assays may improve accessibility and participation, but their substantially lower sensitivity for advanced precursor lesions remains an important limitation. Microbiome-derived and metabolomic signatures remain largely investigational.

The most credible pathway forward is integrated and decision-specific. Clinical risk assessment may estimate baseline susceptibility, molecular testing may provide evidence of inherited risk or prevalent neoplasia, and computational methods may support integration, procedural enhancement, or longitudinal reassessment. These components should be applied according to the clinical question and decision point, with their relative contribution varying across screening contexts.

## 7. Artificial Intelligence: The Third Layer of Precision Colorectal Cancer Screening

Conventional screening programs rely predominantly on a limited set of variables—principally age and, in selected circumstances, family history or recognized high-risk conditions—to determine eligibility and screening intensity. Precision screening, by contrast, seeks to integrate a much broader range of information, including demographic characteristics, clinical risk factors, quantitative FIT results, molecular biomarkers, genomic alterations, microbiome profiles, endoscopic and imaging findings, histopathology, and longitudinal screening history. Although each source may be informative individually, integrating large, heterogeneous, and potentially nonlinear datasets may exceed the capacity of conventional modeling approaches and unaided clinical interpretation [84,125,126,127].

Artificial intelligence (AI) offers computational methods capable of identifying complex patterns within high-dimensional data and generating individualized risk estimates that can be updated as new information becomes available [84,85,127,128,129]. AI should not, however, be regarded as an additional screening modality. Within the framework proposed in this review, it constitutes the third layer of precision screening: a computational layer that may integrate clinical susceptibility, molecular evidence, and procedural findings into interpretable predictions and decision-support outputs.

AI is intended to complement rather than replace established screening strategies and clinical expertise. Potential applications span multiple stages of the pathway, including identification of individuals at elevated risk or those who have not completed screening, support for modality selection, real-time lesion detection and characterization during colonoscopy, and refinement of post-screening surveillance. Its clinical value depends not only on algorithmic performance, but also on transparent reporting, external validation, prospective clinical evaluation, assessment of bias and applicability, and evidence that its use improves outcomes relevant to patients and healthcare systems [128,129,130,131,132].

The most mature application is currently computer-aided lesion detection during colonoscopy, which has been evaluated in multiple randomized trials [17,133]. Broader applications—including CRC risk prediction from routinely collected data, integration of multimodal biomarker panels, and individualized longitudinal risk reassessment—are expanding but remain less clinically established [84,125,126,127]. AI is therefore evolving from a predominantly image-analysis application toward a broader decision-support role across the precision-screening continuum.

### 7.1. From Conventional Prediction to Artificial Intelligence

Artificial intelligence may combine heterogeneous clinical, molecular, endoscopic, pathological, and longitudinal data to generate risk estimates or decision-support outputs that would be difficult to derive from any single source [84,85,125]. Its principal potential advantage lies in identifying complex and potentially nonlinear relationships across multiple variables, particularly when the number, type, and temporal structure of predictors exceed what can be represented readily using prespecified conventional models.

AI should not, however, be assumed to be inherently superior to traditional statistical methods. When predictors are limited, relationships are clinically interpretable, and the intended decision is clearly defined, well-specified regression models may perform equally well while offering greater transparency and simpler implementation. The choice between machine-learning and conventional approaches should therefore be determined by the clinical question, data structure, sample size, interpretability requirements, calibration, external validity, and demonstrated incremental value rather than by computational complexity alone [129,130,131,132].

The clinically useful output is not merely a prediction score. An AI system must link an estimated probability to a predefined decision, such as earlier screening assessment, selection of a particular test, prioritization of diagnostic colonoscopy, or adjustment of surveillance intensity. Its intended population, predicted outcome, time horizon, threshold, and recommended action must therefore be specified explicitly. Without these elements, even a model with apparently strong discrimination may remain difficult to evaluate or incorporate safely into practice.

AI is consequently best understood as a computational and decision-support layer within precision CRC screening rather than as an independent screening modality. Its role is to integrate information, update estimates when clinically meaningful new data become available, and support clinical judgment—not to replace established screening tests, explicit clinical pathways, or human responsibility.

### 7.2. Artificial Intelligence Before Screening

AI may contribute before formal screening begins by identifying individuals who warrant earlier screening assessment or more intensive risk evaluation using routinely collected clinical, laboratory, and electronic health-record data [84,134,135,136]. Machine-learning models combining demographic, metabolic, laboratory, and screening-history information have shown promising predictive performance and may identify individuals whose estimated risk exceeds that suggested by age alone [84,127,134,135,136]. These approaches should be viewed as extensions of established clinical prediction rather than as replacements for it.

Tools based on routinely collected laboratory data, such as ColonFlag, may similarly identify individuals in routine care who warrant further CRC risk assessment [135]. However, their intended clinical role, appropriate referral thresholds, and effects on screening uptake, diagnostic completion, and patient outcomes still require prospective evaluation.

At the population level, predictive analytics may also help identify groups at increased risk of non-participation, allowing screening programs to target reminders, patient navigation, or adherence support more efficiently. This remains a largely investigational application and should not be implemented routinely without prospective evidence that it improves participation and downstream outcomes without reinforcing existing disparities.

### 7.3. Artificial Intelligence During Screening

Once screening begins, AI may contribute at several interconnected stages, including refinement of risk estimates, interpretation of molecular signals, and real-time assistance during colonoscopy. These applications differ substantially in maturity: AI-assisted polyp detection has already been evaluated in numerous randomized trials, whereas multimodal risk-integration models remain predominantly at the development and validation stages [84,95,125,137].

Information generated during screening—including quantitative FIT values, previous screening results, clinical risk factors, and molecular findings—can substantially modify the estimated probability of advanced colorectal neoplasia. AI-based models may eventually combine these data to identify individuals who warrant earlier diagnostic evaluation. For example, a fecal hemoglobin concentration immediately below the conventional FIT positivity threshold may not confer the same risk as an undetectable concentration, illustrating the information lost when a continuous measurement is reduced to a binary result [138,139]. Decision-curve analysis may help determine whether alternative referral thresholds provide clinical net benefit, although findings derived from symptomatic populations may not be directly transferable to asymptomatic screening cohorts [140].

Multitarget stool assays, cell-free DNA tests, and microbiome-derived classifiers may contain multiple interdependent features that cannot be represented adequately by a single biomarker cutoff [95,141,142,143,144]. AI may help translate these heterogeneous signals into clinically interpretable risk estimates or classifications. However, high apparent accuracy in a retrospective case–control dataset does not establish performance in an asymptomatic screening population, where disease prevalence is lower and precursor lesions remain a central target [112].

AI-assisted colonoscopy is currently the most clinically mature application. Computer-aided detection systems analyze the endoscopic video stream continuously and alert the endoscopist to possible lesions. Meta-analyses have consistently shown that computer-aided detection increases adenoma and polyp detection during colonoscopy. The most recent synthesis of 28 randomized trials involving 23,861 participants reported a 20% relative increase in adenoma detection rate and a substantial reduction in adenoma miss rates [17]. Earlier meta-analytic evidence likewise demonstrated a significant increase in adenoma detection with AI-assisted colonoscopy [145]. The incremental benefit appears to arise predominantly from increased detection of small and diminutive adenomas, and direct evidence that computer-aided detection (CADe) reduces post-colonoscopy CRC incidence or mortality remains unavailable [133,137,146].

Computer-aided diagnosis addresses a different task by estimating lesion histology from endoscopic appearance, with meta-analytic evidence showing promising accuracy for the optical characterization of colorectal polyps [137,146,147]. False-positive alerts may distract the endoscopist, while automation bias may lead users to over-rely on highlighted findings or to give insufficient attention to lesions not identified by the system. AI should therefore function as a second observer and decision-support tool, with the endoscopist retaining responsibility for systematic mucosal inspection, lesion characterization, and clinical management [137,146].

### 7.4. Artificial Intelligence After Screening

The contribution of AI does not end with the screening examination. Colonoscopy findings, histopathology, and subsequent screening results generate new information that may modify future risk, and longitudinal models illustrate how estimates may be updated iteratively rather than treating each screening episode as an isolated event [148,149].

Current surveillance recommendations rely primarily on lesion number, size, histological type, and grade of dysplasia [62]. These practical criteria necessarily simplify a broader set of determinants, because individuals with similar colonoscopic findings may still differ in age, family history, metabolic health, examination quality, and adherence—factors that may influence the risk of metachronous neoplasia or post-colonoscopy CRC [149,150,151].

Such longitudinal data are well suited to computational approaches capable of repeated recalibration. Future AI systems may help identify individuals at increased risk of metachronous neoplasia or post-colonoscopy CRC despite apparently reassuring findings and may support structured follow-up for patients at greatest risk of incomplete surveillance [150,151,152,153]. However, much of the current evidence derives from conventional prediction models or retrospective studies rather than prospectively validated AI-guided pathways. These applications therefore remain developmental and require external validation and evidence of improved clinical outcomes before routine adoption [129,130,131,132].

### 7.5. Challenges and Responsible Implementation

Successful implementation depends on substantially more than algorithmic performance. Generalizability remains a major challenge because models developed in selected populations may perform less well in other settings. Development in diverse datasets should therefore be followed by independent external validation in the intended population [129,130,131,132].

Clinicians must understand the population for which a model was developed, the outcome it predicts, the threshold applied, and the recommended response, even when the underlying methodology is complex [131]. Ongoing performance monitoring is also essential because patient populations, data quality, and clinical practice may change over time. Without such oversight, model performance may deteriorate, and implementation may reinforce rather than reduce existing disparities [131,132].

Implementation must also reflect healthcare-system capacity. In settings where organized screening, FIT processing, diagnostic colonoscopy, and treatment access remain limited, strengthening these core services may provide greater population benefit than introducing advanced computational infrastructure [154,155,156].

Workflow integration represents another major challenge. AI tools must provide timely, interpretable, and actionable outputs without creating excessive burden or encouraging automation bias. Evaluation should extend beyond discrimination alone to include calibration, clinical net benefit, screening participation, diagnostic completion, advanced colorectal neoplasia yield, unnecessary procedures, interval CRC, cost-effectiveness, and equity.

### 7.6. Clinical Implications

AI is best regarded as the computational layer of precision screening rather than as an independent screening modality. The strongest current evidence supports AI-assisted colonoscopy, particularly computer-aided lesion detection. Multimodal prediction and individualized surveillance remain promising but require external validation and prospective comparison with existing strategies before implementation.

Near-term adoption will probably involve gradual integration rather than wholesale replacement of established pathways. Success should be judged not by algorithmic complexity, but by whether AI improves accuracy, efficiency, equity, and clinically relevant outcomes across the continuum of CRC prevention.

## 8. Integrating Risk, Biomarkers, and Artificial Intelligence into Precision-Screening Pathways

The transition toward precision colorectal cancer screening depends not on any single superior test, biomarker, or algorithm, but on the coordinated use of information with different clinical functions. Clinical risk factors, genetic susceptibility, molecular biomarkers, screening-test results, endoscopic findings, and computational tools do not provide interchangeable information: some estimate future susceptibility, some detect signals associated with prevalent neoplasia, some improve lesion recognition during an established procedure, and others may support longitudinal reassessment after screening. A precision-screening framework must therefore integrate these sources according to the clinical question, predicted outcome, time horizon, and decision point, rather than simply combine them into a single composite risk estimate.

The three-layer framework proposed in this review should consequently be understood as a conceptual organizational architecture rather than a validated mandatory sequence. Its purpose is to distinguish three complementary functions: clinical risk stratification establishes background susceptibility and may inform entry into screening or selection among appropriate strategies; molecular biomarkers provide additional biological information, ranging from inherited susceptibility to signals associated with prevalent colorectal neoplasia; and artificial intelligence provides computational support for integrating heterogeneous information, assisting procedural detection and characterization, and updating estimates as longitudinal data accumulate. The relative contribution of each layer will vary according to the screening context, and not every individual will require all three layers.

This distinction also defines the framework’s novelty. Multimodal approaches combining clinical, genetic, molecular, or computational information have already been investigated in risk-stratified screening research [86], including models that integrate clinical, proteomic, and polygenic information [87]. The proposed framework does not claim novelty from combining these data types per se. Rather, it organizes them according to their clinical function and position along the screening continuum, explicitly separating susceptibility estimation, detection of prevalent neoplasia, procedural enhancement, and longitudinal reassessment. It further links each function to a potential clinical action while distinguishing established screening practice from emerging or investigational applications. The objective is therefore not maximal technological integration, but selective addition of information when it provides actionable incremental value over an effective population-based pathway.

For clarity, the framework is intended primarily for asymptomatic individuals undergoing CRC screening. Patients with symptoms suggestive of colorectal disease require diagnostic evaluation rather than screening, while individuals with established hereditary cancer syndromes, inflammatory bowel disease, or previous advanced colorectal neoplasia should continue to follow dedicated high-risk screening or surveillance pathways. Similarly, post-polypectomy surveillance represents a distinct clinical context in which previous endoscopic and pathological findings, rather than baseline population risk alone, determine management.

### 8.1. From Parallel Tools to an Integrated Architecture

Clinical risk stratification, molecular biomarkers, and AI are frequently studied as separate domains, but their greatest potential value lies in addressing different questions within the same screening continuum. The first question is **susceptibility**: what is the individual’s probability of developing or harboring clinically significant colorectal neoplasia before a screening test is performed? Routinely available demographic, familial, lifestyle, metabolic, and prior-screening information provides the most accessible starting point for this assessment [41,86].

The second question is **evidence of prevalent neoplasia**. Established screening tests such as FIT and multitarget stool assays detect biological signals associated with an existing lesion rather than merely background susceptibility. Emerging blood-, stool-, proteomic-, or microbiome-based biomarkers may add complementary information, but their interpretation depends on their specific biological target and intended use. A polygenic risk score, for example, estimates inherited susceptibility and should not be interpreted as evidence that a lesion is currently present. Conversely, a positive established stool-based screening test represents an indication for diagnostic evaluation rather than simply another variable to be averaged with clinical risk.

The third function is **integration and decision support**. AI may combine high-dimensional or longitudinal information when clinically justified, but it should not be regarded as a universal final arbiter of screening decisions. Depending on the application, computational methods may support risk estimation before screening, interpretation of multimodal signals, lesion detection or characterization during colonoscopy, or reassessment after screening. Computer-aided detection during colonoscopy is therefore conceptually different from an AI model estimating future CRC risk: the former enhances lesion recognition during an established procedure, whereas the latter may influence a pre-screening decision.

A multimodal UK Biobank analysis illustrates one potential form of integration: addition of proteomic and polygenic scores to a clinical risk model improved prediction and broadened risk separation [87]. Such improvement demonstrates incremental predictive information, but does not itself establish that biomarker-guided screening improves participation, advanced colorectal neoplasia detection, CRC incidence, or mortality. The transition from better prediction to better screening therefore requires prospective evaluation of the management action triggered by the integrated estimate.

Discordant findings require interpretation according to the clinical role of each component rather than automatic averaging across layers. In particular, a positive result from an established non-colonoscopic screening test should prompt diagnostic colonoscopy and should not be downgraded by a lower clinical risk score, AI prediction, or subsequent negative investigational assay. Discordance among emerging biomarkers, susceptibility estimates, or computational outputs may justify repeat assessment or additional validation only within predefined investigational pathways. Thus, integration should preserve established diagnostic obligations while allowing emerging tools to refine decisions where no guideline-mandated action has already been triggered.

Within this architecture, integration should remain **decision-specific rather than technology-driven**. Clinical risk may help determine when screening should begin or which guideline-supported modality is most appropriate; a non-invasive screening result may determine whether diagnostic colonoscopy is required; AI may improve lesion recognition once colonoscopy is performed; and screening, endoscopic, and pathological findings may subsequently inform longitudinal reassessment. Each layer therefore contributes at defined points rather than operating as an unrestricted sequence in which one result can automatically override another.

### 8.2. A Decision-Specific Precision-Screening Pathway

A practical precision-screening pathway should organize available information according to the clinical decision being addressed rather than require every individual to progress through a fixed sequence of increasingly complex tests (Figure 2). Baseline clinical assessment may help identify eligibility, established high-risk conditions, and appropriate screening options; non-colonoscopic screening tests may subsequently determine whether diagnostic colonoscopy is required; and endoscopic, pathological, and longitudinal information may guide later screening or surveillance decisions. The contribution of each component depends on its intended function, evidence base, and incremental value within the existing screening pathway [41,86].

The first decision point involves identification of the screen-eligible population and baseline risk assessment, with individuals who have hereditary CRC syndromes, inflammatory bowel disease, or previous advanced precursor lesions or CRC directed to dedicated high-risk pathways [157]. A second decision point may introduce non-invasive testing—such as FIT, stool DNA testing, or a blood-based assay—selected according to its intended role, the individual’s risk profile, patient preference, and healthcare-system capacity [13,14].

A third decision point translates the estimated probability of clinically significant neoplasia into a predefined action. AI may assist when the available information is high-dimensional or longitudinal, although conventional models may remain preferable when predictors are limited and relationships are readily interpretable [87,96,130,158]. A fourth decision point provides diagnostic resolution through colonoscopy, because failure to complete diagnostic evaluation after a positive or high-risk result would negate much of the benefit of the preceding decision points. A fifth decision point involves longitudinal reassessment using subsequent screening, endoscopic, histopathological, and clinical information [148,149,150,151,152,153].

Such a model would permit incremental adoption within existing screening programs. Excessive fragmentation should nevertheless be avoided. Each additional component may increase complexity, reduce participation, and create new opportunities for loss to follow-up. Every added layer should therefore demonstrate sufficient incremental clinical value to justify its cost, burden, and operational complexity [86,130].

Viewed in this way, precision screening functions as a decision-specific architecture in which additional information is introduced only when it can meaningfully alter a defined clinical decision. More complex, costly, or invasive components should therefore be used selectively when their incremental value justifies the associated burden and resource use.

### 8.3. Matching Screening Intensity to Individual Risk

Integrated risk assessment has clinical value only when it leads to proportionate differences in management. Screening initiation represents one of the most direct opportunities for adaptation. Risk-modeling studies combining lifestyle, environmental, and genetic factors have shown differences of more than a decade in the estimated age at which individuals reach the same predefined CRC-risk threshold [53,159]. However, individualized starting ages derived from such models have not yet been shown in randomized trials to improve CRC incidence or mortality compared with established population-based programs.

Screening modality may also be adapted according to estimated risk, previous screening history, patient preference, and healthcare-system capacity. Individuals at lower estimated risk may be managed initially with accessible non-invasive tests, whereas those at higher risk may warrant a more sensitive first-line strategy or consideration of primary colonoscopy. Higher estimated risk should not, however, automatically mandate colonoscopy if a staged non-invasive pathway can achieve comparable clinical benefit with greater participation and lower procedural burden.

This principle is particularly relevant in FIT-based programs, where fecal hemoglobin concentration provides more information than a binary positive or negative result. A large study found that fecal-hemoglobin-guided screening intervals could reduce the projected number of tests and colonoscopies while maintaining effectiveness comparable to uniform biennial FIT [160]. This supports quantitative refinement of screening intervals, although long-term prospective confirmation remains necessary. Modeling studies similarly suggest that risk-adapted combinations of thresholds and intervals may improve the balance among benefit, burden, and colonoscopy use compared with uniform strategies [161]. Optimization in a model, however, does not by itself establish clinical effectiveness.

Screening intensity must also account for competing risks and capacity to benefit. In older adults or individuals with severe comorbidity, decisions to continue or discontinue screening should incorporate life expectancy, health status, prior screening history, and patient preference rather than chronological age alone [157].

Equity remains an explicit consideration. Healthcare utilization and access may partly reflect structural disadvantage rather than biological risk. Reduced screening intensity should therefore be recommended only when there is credible evidence of lower expected benefit, not merely because an individual has fewer recorded encounters, incomplete data, or limited access to care. Screening intensity should ultimately be guided by expected clinical benefit rather than predicted risk alone.

### 8.4. Dynamic Risk Reassessment Across the Screening Continuum

Baseline stratification provides only an initial estimate. As individuals progress through repeated screening rounds, new information may substantially modify risk. Precision screening should therefore treat reassessment as a longitudinal and iterative process rather than as a one-time assignment.

A negative FIT provides reassurance for the current screening round, while quantitative fecal hemoglobin concentration and previous screening results may further refine subsequent risk and screening intervals [160]. Screening history also carries predictive information. Consistent participation with repeated negative results differs meaningfully from irregular attendance accompanied by prolonged unscreened intervals [149].

Colonoscopy and histopathology provide another opportunity for risk updating. Evidence from serial post-polypectomy surveillance indicates that the yield of a later examination depends on the combined pattern of findings across successive rounds rather than solely on the most recent result [162]. Estimated clinical risk may likewise change as metabolic health, family history, medication use, and previous findings evolve.

Molecular biomarkers may also be suitable for longitudinal interpretation, but assay-specific variability and thresholds for clinically meaningful change must first be established. Without such standards, repeated testing may amplify biological or analytical noise rather than improve prediction.

AI may support dynamic reassessment by integrating newly available clinical, molecular, endoscopic, and pathological information. Its useful output, however, is not simply a frequently updated score. It should provide an updated risk estimate linked to a predefined decision point and should be recalculated only when clinically meaningful new information becomes available.

Communication is equally important. Changes in risk category should be presented as an expected consequence of accumulating information rather than as evidence that the previous assessment was necessarily incorrect. Reassessment must also remain clinically bounded so that minor fluctuations do not trigger unnecessary colonoscopy, repeated testing, or excessive alert burden.

Taken together, longitudinal precision screening transforms the screening record into an accumulating body of evidence and represents a shift from fixed schedules toward adaptive decision-making. Such an approach is credible only when grounded in validated thresholds, explicit clinical actions, equitable implementation, and continued human oversight (Figure 3).

### 8.5. Equity, Feasibility, and Implementation

The clinical value of precision screening will depend not only on predictive performance, but also on accessibility, affordability, participation, and healthcare-system capacity. Approaches that rely on comprehensive electronic records, genetic profiling, advanced molecular assays, or computational infrastructure may perform well in selected populations yet remain difficult to implement equitably at scale. This is particularly relevant in underrepresented populations, where risk models and polygenic scores may show reduced transportability, and in resource-limited settings, where establishing organized screening, FIT processing, diagnostic colonoscopy, and access to treatment may provide greater benefit than introducing additional technological complexity [78,79,80,154,155,156].

Precision screening should therefore be implemented incrementally, with each added layer required to demonstrate clinically meaningful benefit relative to its cost, burden, and potential effect on participation. A simple, accessible pathway should remain available whenever more individualized assessment is unavailable, impractical, or insufficiently validated.

The next phase of precision CRC screening should therefore focus less on developing additional predictors and more on testing whether specific risk-informed actions improve outcomes compared with optimized existing screening. Priority evaluations should specify the intervention, comparator, clinical decision triggered by the precision component, and outcomes assessed at the level of the invited population rather than only among participants. Relevant outcomes include participation, completion of diagnostic colonoscopy, advanced colorectal neoplasia yield per person invited, interval CRC, procedure-related harms, resource use, cost-effectiveness, and performance across clinically and demographically relevant subgroups. Table 3 summarizes several comparisons that could be evaluated prospectively with currently available technologies.

## 9. Conclusions and Future Perspectives

Colorectal cancer screening has traditionally relied on age-based eligibility, standardized intervals, and broadly uniform pathways. These strategies have generated substantial public-health benefit and should remain the foundation of organized screening. However, individuals of the same age do not necessarily share the same susceptibility to CRC, probability of harboring advanced colorectal neoplasia, likelihood of completing screening, or capacity to benefit from more intensive testing. The increasing availability of clinical, molecular, endoscopic, and computational information therefore creates an opportunity to refine screening decisions beyond age alone.

Clinical risk stratification, molecular biomarkers, and artificial intelligence should be regarded as complementary but functionally distinct components of precision screening. Clinical information estimates background susceptibility; molecular data may reflect inherited predisposition or biological signals associated with prevalent neoplasia; and AI may support multimodal integration, procedural enhancement, and longitudinal reassessment. These components should therefore be deployed selectively according to the decision being supported.

The practical value of precision screening lies in applying the appropriate type of information at the decision point where it can change management. Clinical risk may inform screening initiation or modality selection; established non-invasive screening results may determine the need for diagnostic colonoscopy; AI may improve lesion detection and characterization during colonoscopy; and screening, endoscopic, pathological, and clinical information may subsequently refine future screening or surveillance decisions. Once an established screening result mandates diagnostic evaluation, lower clinical risk or a negative investigational result should not override that obligation.

The central translational challenge is therefore not simply to develop more predictors, biomarkers, or algorithms, but to determine whether their use leads to better decisions and better outcomes than optimized established screening. Many approaches reviewed here improve discrimination, diagnostic accuracy, or risk separation, but relatively few have demonstrated improvements in participation, diagnostic completion, advanced colorectal neoplasia yield, interval CRC, harms, resource use, equity, cost-effectiveness, or long-term cancer outcomes. Prospective comparative studies should therefore evaluate not only model performance, but also the clinical action triggered by the model and its consequences at the population level.

Equity and feasibility must remain central to implementation. Precision screening should enhance rather than restrict access to effective CRC prevention, and evidence-based population screening should remain available whenever individualized strategies are unavailable, impractical, insufficiently validated, or unlikely to provide meaningful incremental benefit. Implementation should also remain sensitive to local screening infrastructure, colonoscopy capacity, costs, and the ability to ensure timely diagnostic follow-up.

The most realistic future is therefore not wholesale replacement of population-based screening, but selective integration of validated precision tools where they provide actionable incremental value. The success of this transition should be judged not by technological complexity, but by whether it improves prevention, participation, efficiency, equity, and clinical outcomes compared with established care. The strategy ultimately adopted should be the least complex approach that reliably improves upon effective population-based screening while preserving its accessibility and public-health strengths.

## Figures and Tables

**Figure 1:**
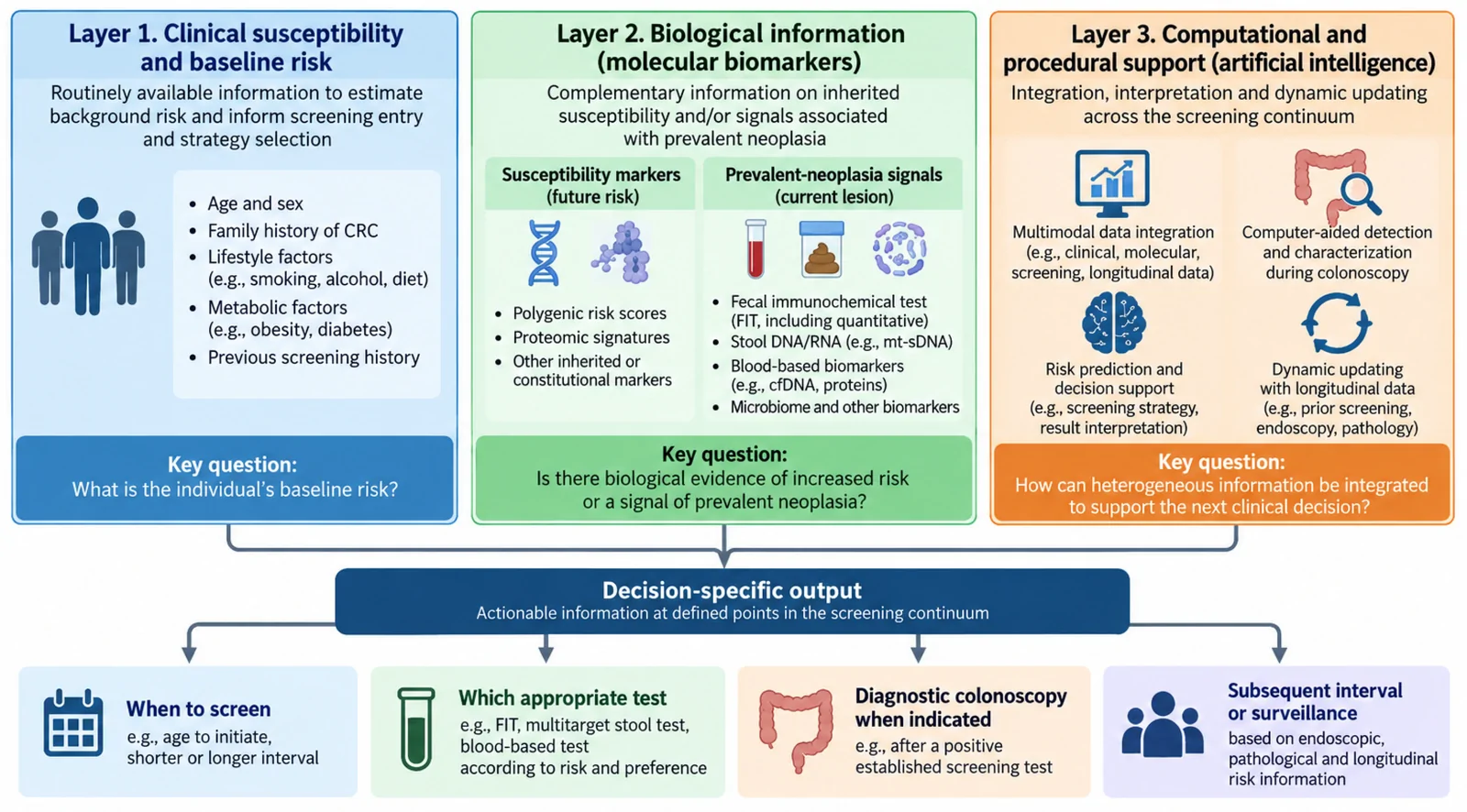
Functional three-layer framework for precision colorectal cancer screening. Clinical information estimates baseline susceptibility; molecular information may reflect inherited susceptibility or signals associated with prevalent neoplasia; and artificial intelligence may support multimodal integration, procedural enhancement, and longitudinal updating. The three layers represent complementary clinical functions rather than a mandatory sequential pathway and are applied according to the clinical question and decision point. Abbreviations: AI, artificial intelligence; CRC, colorectal cancer; FIT, fecal immunochemical test. Image generated using Canva (Canva Pty Ltd., Sydney, Australia; www.canva.com, accessed on 19 September 2026).

**Figure 2:**
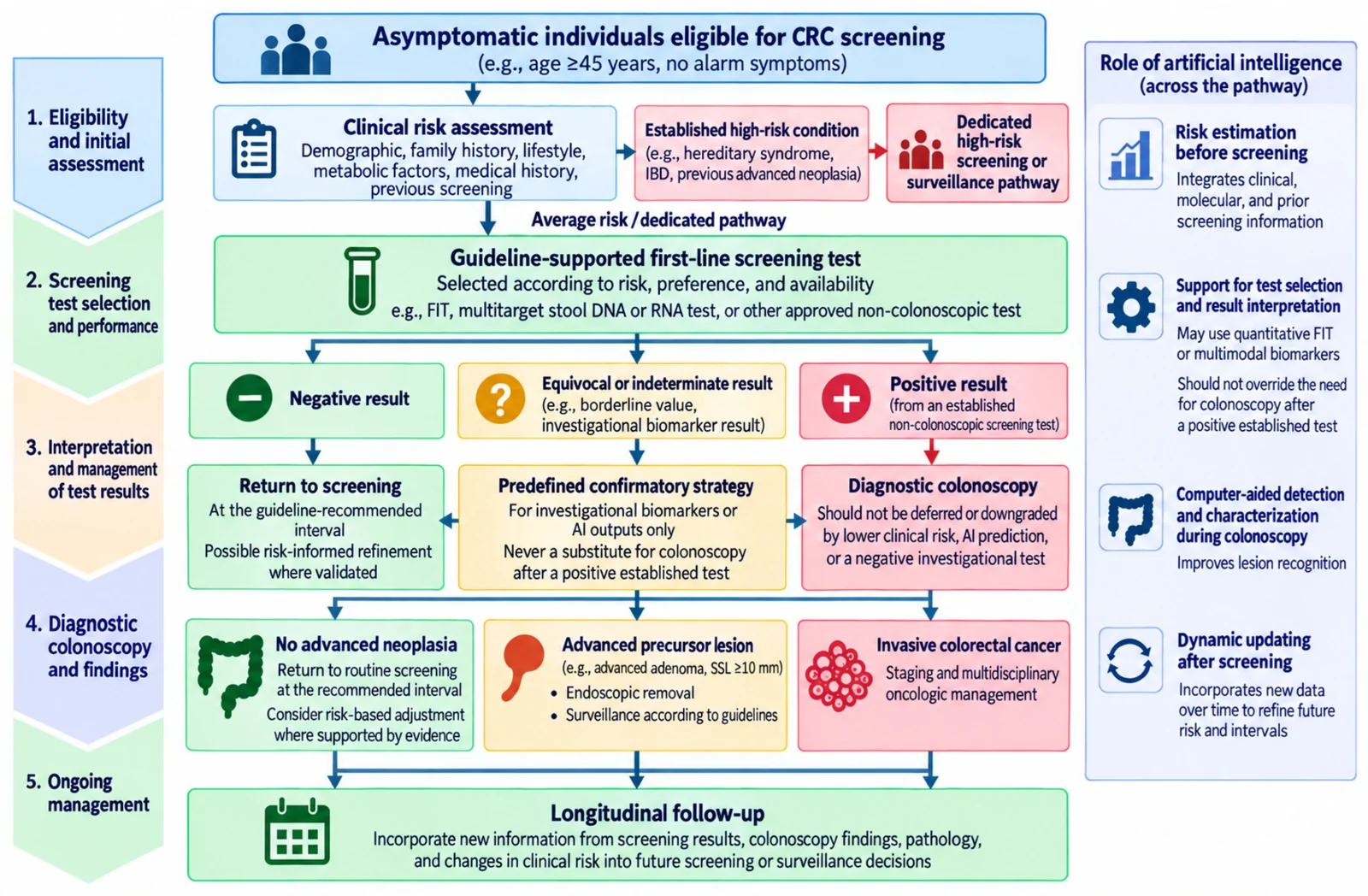
Practical precision-screening pathway for asymptomatic individuals eligible for colorectal cancer screening. Initial assessment identifies individuals who require dedicated high-risk screening or surveillance and directs others toward guideline-supported screening. A positive established non-colonoscopic screening test requires diagnostic colonoscopy and should not be downgraded by a lower clinical risk estimate, artificial-intelligence prediction, or subsequent negative investigational test. Colonoscopy findings subsequently determine return to screening, post-polypectomy surveillance after removal of advanced precursor lesions, or staging and oncologic treatment for invasive CRC. Artificial intelligence may provide decision support at defined points across the pathway but does not replace established diagnostic obligations. Abbreviations: AI, artificial intelligence; CRC, colorectal cancer; FIT, fecal immunochemical test; IBD, inflammatory bowel disease; SSL, sessile serrated lesion. Image generated using Canva (Canva Pty Ltd., Sydney, Australia; www.canva.com, accessed on 19 September 2026).

**Figure 3:**
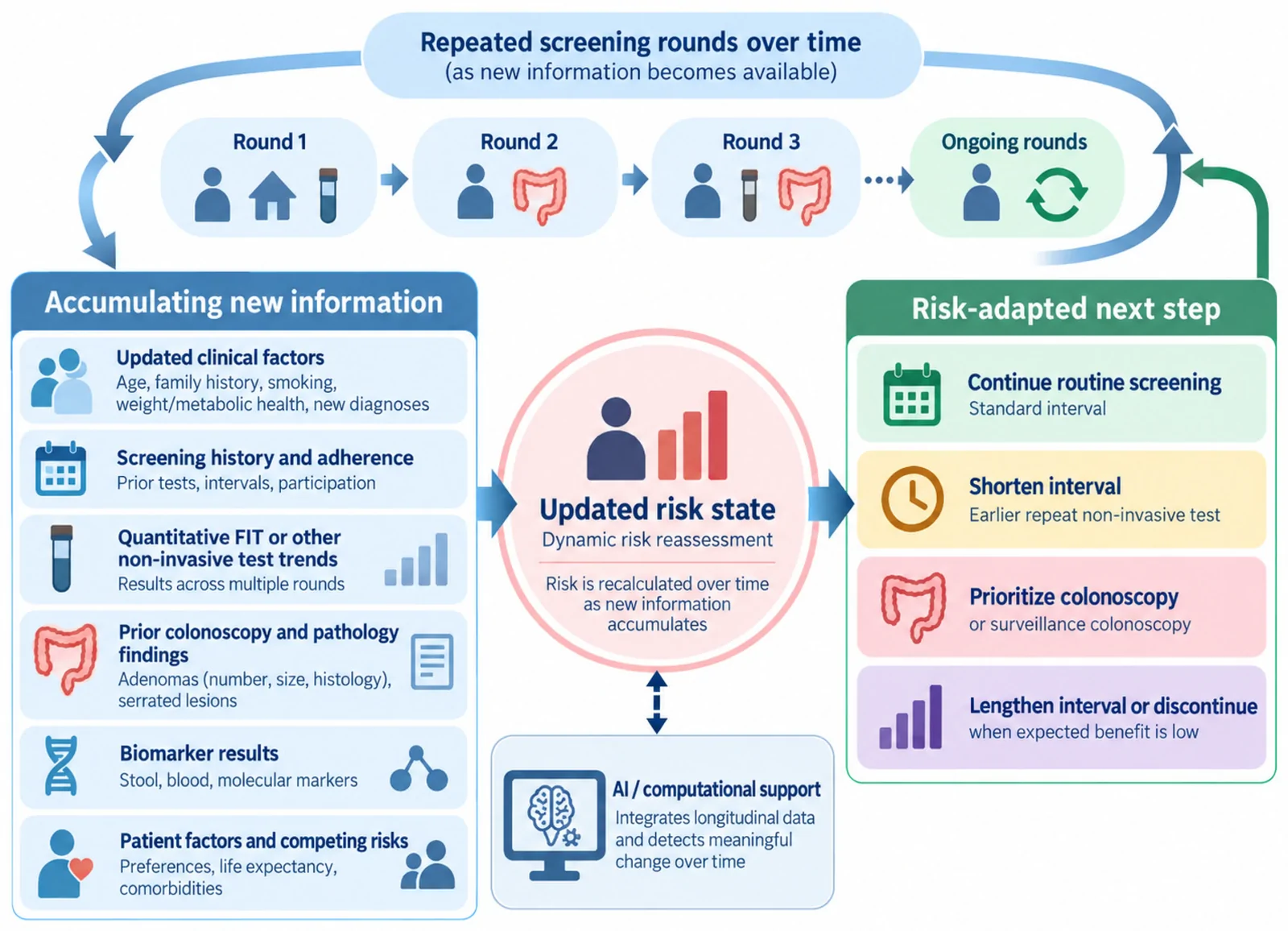
Longitudinal risk reassessment across repeated colorectal cancer screening and surveillance rounds. New clinical information, prior screening participation and results, quantitative or molecular test findings, and previous endoscopic and pathological findings may update an individual’s risk state over time. The updated risk estimate should be linked to a predefined subsequent decision, such as maintaining the recommended interval, shortening or lengthening the interval where validated, or selecting an appropriate screening or surveillance strategy. Artificial intelligence may support integration of longitudinal information but does not itself determine management. Abbreviations: AI, artificial intelligence; CRC, colorectal cancer; FIT, fecal immunochemical test. Image generated using Canva (Canva Pty Ltd., Sydney, Australia; www.canva.com, accessed on 19 September 2026).

**Table 1:** Comparative Characteristics and Current Clinical Roles of Established Colorectal Cancer Screening Modalities.

Screening Modality	Detection Principle	Major Strengths	Principal Limitations	Current Clinical Role
**Guaiac-based fecal occult blood test (gFOBT)** [4,19,20]	Detection of the pseudoperoxidase activity of heme in stool	Inexpensive and non-invasive; supported by randomized evidence demonstrating reduced CRC mortality	Not specific for human hemoglobin; affected by dietary and medication factors; generally requires multiple samples; limited sensitivity for CRC and advanced precursor lesions	Historically important population-based screening test; now largely replaced by FIT where appropriate laboratory infrastructure is available
**Fecal immunochemical test (FIT)** [28,29,30,31]	Immunochemical detection and quantification of human hemoglobin in stool	Non-invasive and inexpensive; requires no dietary restriction; automated and scalable; generally associated with higher participation than invasive tests; permits adjustment of the quantitative positivity threshold	Requires regular repetition; lower sensitivity for advanced precursor lesions than for CRC; depends on intermittent lesion bleeding; reduced detection of some right-sided and serrated lesions	Preferred first-line test in many organized screening programs; positive results require timely diagnostic colonoscopy
**Multitarget stool DNA test** [12,13]	Combined detection of fecal hemoglobin and neoplasia-associated DNA methylation and mutation markers	Higher single-round sensitivity than FIT for CRC and advanced precursor lesions; non-invasive; detects molecular abnormalities in addition to bleeding	Lower specificity than FIT; more false-positive results and diagnostic colonoscopies; higher cost; more complex sample collection and processing; requires repeated testing	Alternative non-invasive screening option where cost, laboratory infrastructure, and colonoscopy capacity permit
**Multitarget stool RNA test (mt-sRNA)** [32,33,34]	Combined detection of stool RNA-transcript markers and fecal hemoglobin, with algorithmic integration of assay and clinical information	Non-invasive; higher sensitivity for CRC and advanced precursor lesions than FIT in validation studies [32,33]; home-based collection; recognized by the 2026 ACS guideline as a preferred stool-based option [34]	Lower specificity than FIT; limited repeated-round and long-term outcome data [33]; greater cost and processing complexity; positive results require diagnostic colonoscopy	Preferred stool-based option every 3 years in the 2026 ACS guideline for average-risk adults in the United States [34]; real-world effectiveness and long-term implementation remain under evaluation
**Colonoscopy** [8,9,24,35,36]	Direct endoscopic visualization of the entire colorectum, with biopsy and polypectomy when required	Detects CRC and precursor lesions; permits diagnosis, tissue sampling, and lesion removal during the same procedure; allows a long interval after a high-quality negative examination	Invasive; requires bowel preparation and often sedation; associated with procedural complications; operator- and quality-dependent; relatively costly and resource-intensive	Primary screening option in selected healthcare systems and the required diagnostic examination after a positive non-invasive screening test
**Flexible sigmoidoscopy** [5,6,23]	Direct endoscopic visualization of the rectum and distal colon	Supported by randomized evidence demonstrating reduced CRC incidence and mortality; requires less preparation and fewer resources than colonoscopy; permits biopsy and distal polypectomy	Does not directly examine the proximal colon; invasive; proximal neoplasia may be missed; positive or high-risk distal findings may require colonoscopy	Evidence-based alternative where program design, resource availability, or patient preference favors limited endoscopy; used less frequently in contemporary programs
**Computed tomographic colonography (CTC)** [8,34,37]	Low-dose CT-based two- and three-dimensional visualization of colorectal structural abnormalities	Does not require sedation; minimally invasive; high sensitivity for CRC and large polyps; useful after incomplete colonoscopy or when conventional colonoscopy is unsuitable	Requires bowel preparation and colonic insufflation; involves ionizing radiation; does not permit biopsy or polypectomy; may detect incidental extracolonic findings; positive results require colonoscopy	Alternative structural examination for individuals who decline colonoscopy, cannot undergo it, or have had an incomplete examination
**Colon capsule endoscopy** [37,38]	Capsule-based optical imaging of the colorectal mucosa	Minimally invasive; requires no sedation or conventional endoscope insertion; provides visualization of the entire colorectal mucosa when the examination is complete	Requires intensive bowel preparation; completion and cleansing quality are variable; does not permit biopsy or treatment; creates a substantial interpretation burden; positive findings require colonoscopy	Selected alternative after incomplete colonoscopy or when conventional colonoscopy is unsuitable or refused, where local expertise and availability permit; not a routine first-line population-screening test

**Note:** ACS, American Cancer Society; CRC, colorectal cancer; CTC, computed tomographic colonography; FIT, fecal immunochemical test; gFOBT, guaiac-based fecal occult blood test; mt-sRNA, multitarget stool RNA. A positive result from any non-colonoscopic screening test requires timely diagnostic colonoscopy. Screening intervals and eligibility criteria vary according to national guidelines, individual risk, test availability, and healthcare-system capacity.

**Table 2:** Representative Clinical Risk-Prediction Models and Model Families Relevant to Colorectal Cancer Screening.

Model or Model Family	Predicted Outcome and Time Frame	Principal Predictors	Development Setting or Population	Reported Performance or Risk Separation	Potential Screening Application	Principal Limitations
Imperiale advanced colorectal neoplasia score [88]	Prevalent advanced colorectal neoplasia at first screening colonoscopy	Age, sex, waist circumference, cigarette smoking, family history of CRC	Multicenter US screening-colonoscopy study; derivation and separate validation cohorts within the same study; not independent external validation	Advanced colorectal neoplasia prevalence 1.92%–24.9% across risk groups in derivation cohort and 1.65%–22.3% in validation cohort	Pre-screening stratification of expected colonoscopy yield	Selected US population; limited predictors; needs external validation/recalibration elsewhere
Asia-Pacific Colorectal Screening score [99]	Prevalent advanced colorectal neoplasia	Age, sex, first-degree family history, smoking status	Asymptomatic adults across Asia-Pacific populations	Clear risk separation, with substantially greater prevalence in high-risk group	Simple pre-screening stratification and prioritization of colonoscopy in resource-constrained settings	Moderate discrimination; calibration and category yields vary by population
External validations of the APCS score [100,101]	Prevalent advanced colorectal neoplasia at screening colonoscopy	Original APCS predictors	Western and Chinese asymptomatic screening populations	Stratification ability retained, though discrimination and category yields varied	Assessing APCS transportability and local colonoscopy prioritization	Performance and category yields varied across populations; recalibration may be required before local implementation.
APCS score combined with stool DNA testing [102]	Prevalent advanced colorectal neoplasia	APCS clinical score plus stool DNA testing	Chinese asymptomatic adults undergoing colonoscopy	Improved detection of advanced colorectal neoplasia compared with either component alone	Two-stage clinical-molecular triage for colonoscopy referral	Added cost/complexity; single-population evidence; limited external/prospective validation
Advanced colorectal neoplasia risk scores in systematic reviews [89]	Prevalent advanced adenoma or neoplasia at screening colonoscopy	Commonly age, sex, family history, smoking, BMI/waist circumference, diabetes	Average-risk screening/colonoscopy populations, multiple countries	Generally modest-to-moderate discrimination, substantial heterogeneity among models	Identification of groups with different expected colonoscopy yields or a potential need for more intensive screening	Heterogeneous outcomes/predictors/thresholds; calibration and utility often underreported
CRC risk-prediction models in systematic reviews [11,55]	Future incident CRC over variable horizons	Demographic, familial, lifestyle, medical, sometimes genetic factors	Cohort, primary-care, and screening populations, multiple countries	Generally modest-to-moderate discrimination, considerable variability	Estimation of future CRC risk and potential adaptation of screening initiation or intensity	Limited external validation; inconsistent calibration reporting; uncertain benefit over age-based screening
Jeon et al. absolute-risk model [53]	Future absolute CRC risk and estimated age at a predefined population-risk threshold	Age, sex, family history, lifestyle/environmental exposures, common genetic variants	Large US case–control and prospective cohort datasets	Estimated starting age varied substantially across combined risk profiles	Individualized screening-initiation age	Requires genetic/detailed risk-factor data; transportability, cost, and clinical benefit remain uncertain
QCancer 10-year bowel-cancer risk algorithm [103]	10-year risk of incident bowel cancer	Age, sex, ethnicity, smoking, alcohol use, family history of bowel cancer, ulcerative colitis, previous colonic polyps, type 2 diabetes, and selected previous cancers	QResearch primary-care database, England; derivation in 565 practices and validation in 188 separate practices within the same QResearch system (separate-practice validation; not independent external validation)	Strong discrimination within the development healthcare system, with performance varying by cancer site and validation population	Automated estimation of future CRC risk from routinely collected primary-care data	Dependent on completeness and coding quality of routine electronic records; developed in UK primary care; requires external validation before use in other healthcare systems; not a symptom-based diagnostic tool
Polygenic and combined clinical-genetic risk models [52,78,79,80]	Future CRC incidence, advanced colorectal neoplasia risk, lifetime risk, or individualized starting age	Polygenic risk scores alone or with clinical/lifestyle factors	Genetic epidemiology and screening cohorts, predominantly European ancestry	Greater risk separation than age or clinical factors alone in several studies	Earlier/more intensive screening for high inherited susceptibility	Reduced transportability across ancestry groups; added cost/complexity; uncertain impact on participation, equity, outcomes
Clinical-proteomic-polygenic multimodal model [87]	Future CRC risk and estimated risk-adapted screening starting age	QCancer-15 clinical score, plasma proteomic score, polygenic score	UK Biobank-based cohort	Incremental improvement in discrimination and wider risk separation vs. clinical score alone	Refining intermediate risk and risk-adapted screening initiation age	Biobank-based evidence; clinical utility not established; complex assays may limit scalability

**Note:** Performance estimates should be compared cautiously: models predict different outcomes, use different horizons and thresholds, and were evaluated in populations with differing baseline risks. Reported discrimination alone does not establish calibration, clinical utility, cost-effectiveness, or improved screening outcomes. **Abbreviations:** APCS, Asia-Pacific Colorectal Screening; BMI, body mass index; CRC, colorectal cancer.

**Table 3:** Priority Evaluation Agenda for Precision Colorectal Cancer Screening.

Proposed Application	Current Evidence	Appropriate Comparator	Key Outcomes Required Before Implementation
Clinical risk score used to modify screening initiation	Prediction and modeling studies; limited prospective impact evidence	Standard age-based initiation	CRC and advanced colorectal neoplasia detection per person invited; participation; colonoscopy burden; interval CRC; harms; subgroup calibration
Clinical risk score plus FIT	Feasibility and risk-adapted screening evidence, including TARGET-C [41]	Optimized guideline-based FIT program	Participation; diagnostic completion; advanced colorectal neoplasia yield; colonoscopies per advanced lesion detected; interval CRC; cost-effectiveness
Quantitative FIT-guided screening intervals	Cohort and modeling evidence	Fixed-interval FIT	Cumulative advanced colorectal neoplasia detection; interval CRC; number of tests; colonoscopy use; repeated-round adherence
Stool molecular testing after clinical risk stratification	Diagnostic accuracy and limited prospective evidence	FIT or established stool-based testing	Incremental detection of advanced precursor lesions; specificity; colonoscopy demand; adherence; cost per advanced lesion detected
Blood-based screening for individuals who decline or do not complete preferred screening	Prospective diagnostic-accuracy evidence; limited outcome evidence	Continued outreach for preferred stool-based or structural screening	Incremental screening participation; completion of diagnostic colonoscopy; advanced colorectal neoplasia and CRC yield; stage distribution; repeated-round adherence
Multimodal clinical–proteomic–polygenic risk prediction	Improved discrimination in cohort studies	Clinical risk model alone	Prospective change in screening decisions; calibration; net benefit; equity across ancestry groups; cost; clinical outcomes
AI-assisted colonoscopy	Randomized evidence for increased adenoma detection	High-quality conventional colonoscopy	Advanced-lesion detection; adenoma miss rate; post-colonoscopy CRC; procedure time; false-positive alerts; harms
AI-guided longitudinal risk reassessment	Retrospective/developmental prediction evidence	Guideline-based surveillance intervals	Metachronous advanced neoplasia; interval CRC; unnecessary colonoscopy; calibration over time; subgroup performance
Risk-based de-intensification	Mainly modeling and observational evidence	Standard screening interval	Missed advanced neoplasia; interval CRC; CRC mortality; adherence; patient acceptability; long-term safety

**Note:** AI, artificial intelligence; CRC, colorectal cancer; FIT, fecal immunochemical test. “Advanced neoplasia” refers to CRC and advanced precursor lesions as defined by the respective study. Proposed applications should be evaluated against optimized established screening rather than against no screening.

## Data Availability

No new data were created or analyzed in this study.

## Supplementary Materials

The following supporting information can be downloaded at https://www.mdpi.com/article/10.3390/medicina62101875/s1, Table S1: Representative search concepts used for the narrative review.

## Abbreviations

The following abbreviations are used in this manuscript:

AI	Artificial intelligence
APC	Adenomatous polyposis coli
APCS	Asia-Pacific Colorectal Screening
AUC	Area under the receiver operating characteristic curve
BMI	Body mass index
CADe	Computer-aided detection
CI	Confidence interval
CRC	Colorectal cancer
CT	Computed tomography
CTC	Computed tomographic colonography
DNA	Deoxyribonucleic acid
FIT	Fecal immunochemical test
gFOBT	Guaiac-based fecal occult blood test
IBD	Inflammatory bowel disease
KRAS	KRAS proto-oncogene, GTPase
MASLD	Metabolic dysfunction-associated steatotic liver disease
mt-sRNA	Multitarget stool RNA
OR	Odds ratio
PROBAST	Prediction model Risk Of Bias ASsessment Tool
RNA	Ribonucleic acid
SEPT9	Septin 9
TP53	Tumor protein p53
TRIPOD	Transparent Reporting of a multivariable prediction model for Individual Prognosis Or Diagnosis
