# Supplementary material for: Toward Precision Colorectal Cancer Screening: Integrating Clinical Risk Stratification, Molecular Biomarkers, and Artificial Intelligence
Source: Medicina (Kaunas). 2026 Sep 28;62(10):1875. doi: 10.3390/medicina62101875 (PMC13707249; doi:10.3390/medicina62101875)
Supplement: Supplementary file 1 [file medicina-62-01875-s001.zip › medicina-4556224-supplementary.pdf]

Supplementary Table S1. Representative Search Concepts Used for the Narrative Review

**Domain**	**Representative search concepts**
General CRC screening	"colorectal cancer" AND screening; "colorectal neoplasia" AND screening; population screening AND colorectal
Clinical risk stratification	colorectal AND screening AND ("risk prediction" OR "risk model" OR "risk stratification"); advanced neoplasia AND prediction
Genetic and polygenic risk	colorectal cancer AND ("polygenic risk score" OR genetic risk) AND screening
Stool biomarkers	colorectal AND screening AND (FIT OR fecal immunochemical test OR stool DNA OR stool RNA OR methylation)
Blood-based biomarkers	colorectal cancer AND screening AND ("cell-free DNA" OR cfDNA OR methylation OR fragmentomics OR circulating DNA OR blood-based screening)
Microbiome/metabolomics	colorectal cancer AND screening AND (microbiome OR microbiota OR metabolomics OR microbial biomarker)
Artificial intelligence	colorectal cancer AND screening AND ("artificial intelligence" OR machine learning OR deep learning OR prediction model)
AI-assisted colonoscopy	colonoscopy AND ("computer-aided detection" OR CADe OR CADx OR artificial intelligence) AND adenoma
Precision screening	colorectal AND ("precision screening" OR personalized screening OR individualized screening OR risk-adapted screening)
Implementation/economics	colorectal screening AND (implementation OR participation OR adherence OR cost-effectiveness OR equity OR capacity)

Note: Search terms were adapted to the syntax and indexing conventions of PubMed, Embase, Scopus, and Web of Science. Searches were targeted rather than designed as an exhaustive systematic-review strategy. Reference lists of relevant guidelines, systematic reviews, and landmark studies were additionally screened. **Abbreviations:** AI, artificial intelligence; CADe, computer-aided detection; CADx, computer-aided diagnosis; cfDNA, cell-free DNA; CRC, colorectal cancer; FIT, fecal immunochemical test.
